# 

**Published:** 1875-03

**Authors:** 


Vol. V. MARCH, 1875. No. 3.

### THE SOUTHERN

### MEDICAL RECORD:

PUBLISHED MONTHLY AT

ATLANTA, GEORGIA.

S.G.O. INDEXED

LOCAL EDITORS:

THOS. S. POWELL, M.D.

W. T. GOLDSMITH, M.D. R. C. WORD, M.D.

ASSOCIATE EDITORS:

T. CURTIS SMITH, M.D., - - - - MIDDLEPORT, OHIO.
SWAN M. BURNETT, M.D., - - - KNOXVILLE, TENNESSEE.
ALBAN S PAYNE M.D., - - - - MARKHAM'S, VIRGINIA.
A. R. KILPATRICK, M.D., - - - NAVASOTA, TEXAS.
A. A. LYON, M.D., - - - - COLUMBUS, MISSISSIPPI.
G. A. BAXTER, M.D., - - - CHATTANOOGA, TENN.
J. B. MATTISON, M.D., - - - - CHESTER, NEW JERSEY.

*"QUICQUID PRÆCIPIES ESTO BREVIS."*

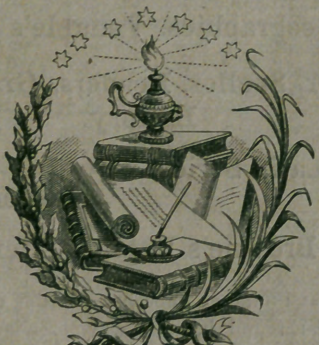

ATLANTA, GEORGIA:
SOUTHERN PUBLISHING COMPANY. PRINTERS.
1875.

TERMS, $3 00 PER ANNUM, IN ADVANCE. SINGLE NUMBER, 35 CENTS.

